# Efficacy of IV immunoglobulins on depressive symptoms and self-injury: A case report

**DOI:** 10.1192/j.eurpsy.2021.2021

**Published:** 2021-08-13

**Authors:** A. Silva, P. Politi, N. Brondino, M. Olivola

**Affiliations:** Department Of Brain And Behavioral Sciences, University of Pavia, Pavia, Italy

**Keywords:** acting-out, immunoglobulins, Borderline, Depression

## Abstract

**Introduction:**

Some studies in literature highlight the correlation between immune-mediated inflammatory processes and psychiatric pathologies. However, there are few studies about the efficacy of IV immunoglobulins in psychiatric features (1). (1) ZUNSZAIN, Patricia A.; HEPGUL, Nilay; PARIANTE, Carmine M. Inflammation and depression. In: Behavioral neurobiology of depression and its treatment. Springer, Berlin, Heidelberg, 2012. p. 135-151.

**Objectives:**

Case report: a 39 year patient diagnosed with borderline personality disorder and myasthenia was hospitalized for self-injury ideation, acting out and depressive episode treated with acid valproic, aripiprazole, gabapentin; flare-up of myasthenia that needed treatment.

**Methods:**

Clinical and test evaluation was performed in three stages: before (t0), immediately after (t1) and 3 weeks after (t2) the administration of the IgEV without other treatment modifications. We have used: - Inventory of Statements About Self-Injury (ISAS) - Barrat Impulsiveness Scale, Version 11 (BIS-11) - Hamilton Anxiety Rating Scale (HAM-A) - Montgomery-Asberg Depression Rating Scale (MADRS) - Alexian Brothers Urge to Selfe-Injure Scale (ABUSI)

**Results:**

The patient has a score of 79 at BIS-11. She used to have a huge number of acting aout as we see on ISAS (Fig.1).

**Figure 1:**
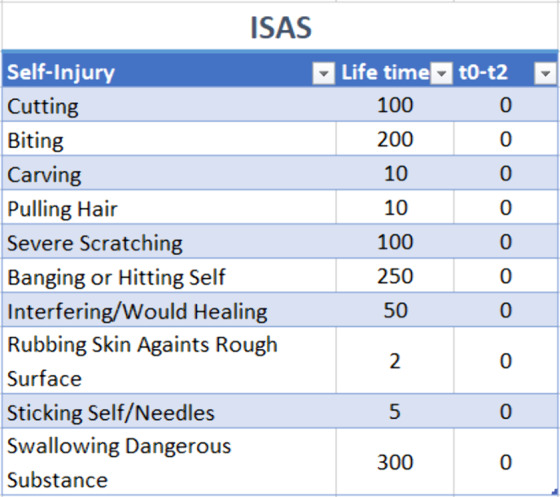

**Figure 2:**
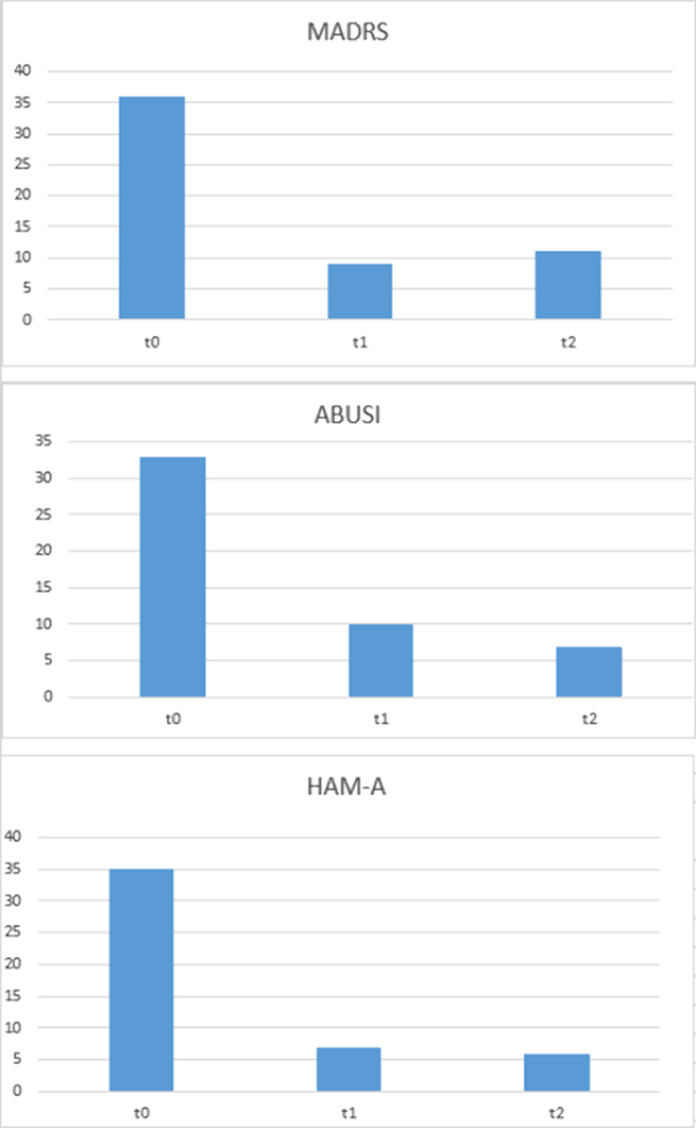

**Conclusions:**

We observed a reduction in non-suicidal self-injurious ideation, the suspension of acting-out, a complete remission of depressive symptoms with mild persistence of anxious symptoms immediately after the administration of immunoglobulins, and the remission continue until one month after the administration (Fig.2).

**Disclosure:**

No significant relationships.

